# Posterior Association Networks and Functional Modules Inferred from Rich Phenotypes of Gene Perturbations

**DOI:** 10.1371/journal.pcbi.1002566

**Published:** 2012-06-28

**Authors:** Xin Wang, Mauro A. Castro, Klaas W. Mulder, Florian Markowetz

**Affiliations:** 1 Cancer Research UK Cambridge Research Institute, Cambridge, Cambridgeshire, United Kingdom; 2 Department of Oncology, University of Cambridge, Cambridge, Cambridgeshire, United Kingdom; University of Chicago, United States of America

## Abstract

Combinatorial gene perturbations provide rich information for a systematic exploration of genetic interactions. Despite successful applications to bacteria and yeast, the scalability of this approach remains a major challenge for higher organisms such as humans. Here, we report a novel experimental and computational framework to efficiently address this challenge by limiting the ‘search space’ for important genetic interactions. We propose to integrate rich phenotypes of multiple single gene perturbations to robustly predict functional modules, which can subsequently be subjected to further experimental investigations such as combinatorial gene silencing. We present posterior association networks (*PANs*) to predict functional interactions between genes estimated using a Bayesian mixture modelling approach. The major advantage of this approach over conventional hypothesis tests is that prior knowledge can be incorporated to enhance predictive power. We demonstrate in a simulation study and on biological data, that integrating complementary information greatly improves prediction accuracy. To search for significant modules, we perform hierarchical clustering with multiscale bootstrap resampling. We demonstrate the power of the proposed methodologies in applications to Ewing's sarcoma and human adult stem cells using publicly available and custom generated data, respectively. In the former application, we identify a gene module including many confirmed and highly promising therapeutic targets. Genes in the module are also significantly overrepresented in signalling pathways that are known to be critical for proliferation of Ewing's sarcoma cells. In the latter application, we predict a functional network of chromatin factors controlling epidermal stem cell fate. Further examinations using ChIP-seq, ChIP-qPCR and RT-qPCR reveal that the basis of their genetic interactions may arise from transcriptional cross regulation. A Bioconductor package implementing PAN is freely available online at http://bioconductor.org/packages/release/bioc/html/PANR.html.

## Author Summary

Synthetic genetic interactions estimated from combinatorial gene perturbation screens provide systematic insights into synergistic interactions of genes in a biological process. However, this approach lacks scalability for large-scale genetic interaction profiling in metazoan organisms such as humans. We contribute to this field by proposing a more scalable and affordable approach, which takes the advantage of multiple single gene perturbation data to predict coherent functional modules followed by genetic interaction investigation using combinatorial perturbations. We developed a versatile computational framework (*PAN*) to robustly predict functional interactions and search for significant functional modules from rich phenotyping screens of single gene perturbations under different conditions or from multiple cell lines. *PAN* features a Bayesian mixture model to assess statistical significance of functional associations, the capability to incorporate prior knowledge as well as a generalized approach to search for significant functional modules by multiscale bootstrap resampling. In applications to Ewing's sarcoma and human adult stem cells, we demonstrate the general applicability and prediction power of *PAN* to both public and custom generated screening data.

## Introduction

An important goal of systems biology is to understand how genes act in concert with each other to control a biological process. Large-scale gene silencing coupled with rich phenotypic screening paves the road towards a systematic understanding of gene functions. Rich phenotypes can result from quantifying many different phenotypic changes in an organism or population of cells [1]–[3] or from observing the same phenotype in different conditions [4], [5]. RNA interference based gene perturbation has been widely used in various organisms, from classic genetic systems such as *C. elegans* and *Drosophila* to higher organisms such as humans [6]. However, knowing the function of the individual gene does not reveal their functional interplay.

Quantitative synthetic genetic interactions evaluated from combinatorial perturbations provide rich information about underlying network structure of biological processes [7]–[10]. For example, combinatorial drug treatments in bacteria and double mutants in yeast have been implemented to explore their underlying cellular networks [9], [11]–[13]. Very recently, RNAi based combinatorial gene silencing was applied to *Drosophila* cell culture for signalling pathway reconstruction [14].

A major limitation of combinatorial gene silencing, however, lies in its scalability in higher organisms such as humans. Genetic interaction profiling requires double knock-down experiments over all possible combinations of RNAi reagents targeting each pair of genes; thus, the very recent application to *Drosophila* cell culture took more than 70,000 pairwise perturbations between only 93 genes involved in signal transduction [14]. This explains why genetic interaction profiling for metazoan genes is still limited to a relatively small scale. Moreover, the quality of RNAi screens may suffer from false positives and false negatives due to a lack of efficacy and specificity in silencing reagents [15], [16]. Meta-data analysis or high quality custom screens are needed to overcome these shortcomings [16], [17]. Instead of combinatorial perturbations, we propose to make efficient use of perturbation data on single genes to predict their functional connections. Our motivation is inspired by the fact that genes that genetically associate very often exhibit correlated phenotypes [9]. Only those coherent modules that are highly functionally connected are then subjected to comprehensive biological analysis for deciphering their synergistic functions in a particular process. Thus, our proposed approach starts from building a large-scale landscape of putative functional interactions and results in a condensed core functional module to prioritise further tests for genetic interactions. This strategy makes it possible to integrate publicly available data sets of single gene perturbations performed across multiple cell lines or under different biochemical conditions.

### Challenges

Our biological strategy poses two key challenges to computation: (a) how to assess the statistical significance of functional interactions computed from phenotyping screens of single gene perturbations; (b) how to integrate complementary data, such as protein-protein interactions, as *a priori* knowledge. Conventional statistical approaches, such as parametric hypothesis tests or permutation based nonparametric methods, often cannot address both challenges efficiently in a joint way. As an alternative, we use a Bayesian mixture modelling approach to simultaneously address both key challenges. We developed a versatile computational framework called *Posterior Association Networks* (*PANs*), which features several main contributions:

- *PAN* uses beta-mixture models as a general framework to infer relevant functional links between genes. The distribution of functional interactions is considered as a mixture of three components representing positive association (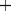), negative association (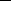) and lack of association (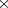), respectively. A beta-mixture model is fitted to the mixed distribution, and posterior probabilities are computed to quantify the statistical significance of each pair of genes having a functional interaction.
- *PAN* allows efficient use of prior information about functional interactions. In our extended beta-mixture model, stratum-specific prior probabilities are set for modelling associations with and without prior evidences. The stratification approach enables integrating potential prior knowledge of functional connections such as protein-protein interactions. We demonstrate in our simulation studies that this extension can make substantial improvement for screening data with a small sample size and weak modularities.
- *PAN* provides a generalized approach to identify statistically significant gene modules. We first perform hierarchical clustering on functional interaction profiles to predict significant gene clusters. The uncertainty of the clustering analysis is assessed by multiscale bootstrap resampling, and an approximate unbiased *p*-value is computed for each cluster to evaluate the significance [18]. Top significant gene clusters are then superimposed onto the inferred posterior association network to obtain functional modules.

### Comparison to other approaches

Previous methods to predict genetic interactions in model organisms have made use of physical interactions [19]–[21] or metabolic networks [22]. Another approach by Lee *et al.* integrates various types of functional genomics data (e.g. coexpression, literature curated protein-protein interaction, gene neighbours, cocitation) to predict functional networks in yeast and *C. elegans* [23]. Our computational framework differs from these approaches by focussing on single-gene perturbation data and integrating them with prior knowledge such as physical interactions. Our application to human epidermal stem cells shows this combination to be very informative. Our approach also differs from predicting genetic interactions by training a network based on known genetic interactions, which may be difficult to be applied to higher organisms such as humans due to a limited number of identified genetic interactions [24]–[26]. Different from network models such as Bayesian networks and extensions (e.g. the random-arcs-and-nodes model [27]) where global optimization is used for inference, *PANs* belong to a large family of networks encoding pairwise association (e.g. correlation coefficients, mutual information and genetic interactions). Deviating from other network models, edges of *PANs* represent posterior beliefs of functional association.

Clustering methods have been used for functional module searching from rich RNAi phenotyping screens [1]–[3]. Different from these multiparametric phenotypes, which requires special feature selection in data preprocessing or distance metric learning techniques, our screening data measures the same phenotype in different conditions. Thus, we adopt hierarchical clustering on functional association profiles with multiscale bootstrap resampling based on *pvclust* [18] to search for significant functional modules. Instead of comparing individual genes' functions, this approach compares functional profiles of genes. A similar strategy has been demonstrated before to be highly desirable to group genes with similar interaction patterns [9].

### Biological strategy

Synthetic genetic interaction profiling lacks scalability to metazoans such as *Drosophila* and humans [14]. Here we propose to integrate single gene perturbation screens to predict their functional interactions. Only those coherent modules that are highly functionally connected are subjected to further investigation for their genetic interactions. This strategy is much more affordable and efficient for systematically studying genes and their synergistic functions in a particular biological process.

The rich phenotyping screens can be obtained from public data sets or custom generated. In the first case study of the paper, the data came from published high-throughput RNAi screens using a kinome siRNA library in four different cancer cell lines [4]. As increasing number of large datasets of genetic screens (e.g. RNAi-based) become available, public databases of gene perturbation screens provide valuable resources. For example, the latest version of the GenomeRNAi database (version 6.0, checked on Feb. 3, 2012) includes 96 RNAi screens in human and 150 screens in *Drosophila* [28]. Computational tools to efficiently mine these data are lacking. Our method is well equipped for this challenge.

**Figure 1:**
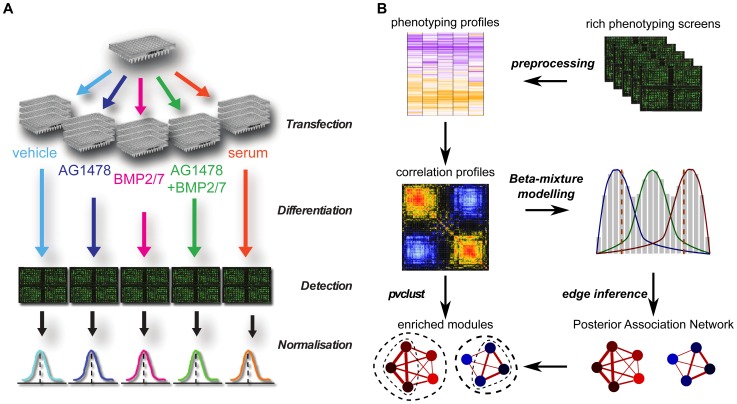
An integrative framework for predicting functional interactions and enriched modules. (A) Experimental strategy. A typical experimental workflow for RNAi screening involves RNAi transfection, different biochemical treatments, reporting phenotypes as well as data preprocessing The schematic figure illustrates how to customize rich phenotyping screens to study epidermal stem cell fate. (B) Computational framework. *PAN* takes as input various types of phenotyping screens (e.g. gene expression, biochemical signals, imaging data of cell morphologies and tissue architectures) that have already been preprocessed. Two parallel sub-workflows are subsequently performed to predict (i) significant functional interactions between genes by beta-mixture modelling on functional association profiles, and (ii) significant gene clusters by hierarchical clustering on functional association profiles. Superimposing the predicted significant gene clusters onto the predicted posterior association network, we finally obtain modules enriched for functional interactions.

In our second application, we generated our own perturbation data to explore functional interactions between chromatin factors in epidermal stem cells. A typical experimental workflow includes RNAi transfection, different biochemical treatments, reporting phenotypes as well as data preprocessing (Figure 1(A)). Specifically in our application, primary human keratinocytes were transfected by an siRNA library targeting 332 potential chromatin-factors. 72 hrs after transfections, cells were treated in five conditions (vehicle, AG1478, BMP2/7, AG1478+BMP2/7 and 10% serum) to induce differentiation for 48 hours. Differentiation status was assessed using an immunofluorescence based assay measuring Endogenous transglutaminase I (TG1) levels. After subtracting background signals, TG1 levels were normalized to control signal to obtain a measure of differentiation per cell. Finally, Z-scores were calculated to standardize the normalized TG1 signals. More details about the experimental design can be found in our accompanying paper [5].

### Overview of this study

We demonstrate the general applicability of our computational methodology on a publicly available data set of single RNAi perturbations across four cell lines in Ewing's sarcoma (ES) [4]. Using the proposed approach, we prioritized one module enriched for confirmed and promising potential therapeutic targets for ES and highly associated with signalling pathways that are known to be critical for proliferation of ES cells. The dense functional connections among genes in the module may imply their genetic interactions, which are worth further biological investigations. In a second in-depth case study, we used *PAN* to infer a functional network of chromatin factors controlling human adult stem cell fate from RNA interference screens in five biochemical conditions. Our approach identified four significant functional modules. Among these modules the one consisting of *ING5*, *UHRF1*, *EZH2*, *SMARCA5*, *BPTF*, *SMARCC2* and *PRMT1* is of particular interest, as it indicates a functional connection between *UHRF1*, *EZH2*, NURF and MORF complexes, which have been independently implicated in epidermal self-renewal [5]. We validated inferred interactions in combinatorial knock-down experiments [5]. Here we show how additional ChIP-seq, ChIP-qPCR and RT-qPCR reveal that the genetic interactions between these five genes may involve transcriptional cross regulations.

## Results

We first describe a unified framework for predicting functional interactions and enriched modules and then assess its power in the controlled setting of a comprehensive simulation study. Finally, we describe novel biological insights made possible by our approach in two case studies: The first one on prioritizing a potential therapeutic network for Ewings sarcoma, and the second one on predicting and confirming a genetic interaction network controlling stem cell fate.

### A unified framework for predicting functional interactions and enriched modules

To represent functional interactions between perturbed genes, we introduce posterior association networks (*PANs*). A posterior association network 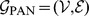 is a type of gene network encoding gene functions on vertices (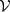) and functional connections between genes on edges (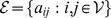). In a *PAN* for genetic screens, each vertex (gene perturbed) is associated with its loss of function quantified by a statistic such as Z-score, whereas each edge encodes *a posteriori* belief in the existence of a functional association between two genes. To predict a *PAN* and functional modules, we developed a unified computational framework (Figure 1(B)) involving the following major procedures:

#### Profiling functional associations

A conventional way to quantify the functional association between two genes is to compute the similarity between their phenotypic profiles based on correlation coefficients (e.g. [1]). Here, we prefer the uncentered correlation coefficient (also known as *cosine similarity*, details in section *Cosine similarity* of Methods), because it considers both magnitude and direction and has been very successful in exploring gene expression patterns [29]–[31]. Thus, we will focus on cosine similarities throughout this manuscript, although other correlation coefficients can be used without changing our methodology.

#### Beta-mixture modeling

Motivated by the density pattern of association profiles, we propose to model functional associations by a mixture of three components representing positive association (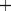), negative association (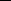) and lack of association (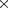), respectively. We employ a stratification strategy to take into consideration potential prior knowledge for the functional network such as protein-protein interactions (details in section *The extended beta-mixture model* of Methods). To fit the beta-mixture model, we performed MAP (maximum *a posteriori*) based on the EM algorithm (details in section *Maximum a posteriori (MAP) inference* of Methods) [32].

#### Network inference

To assess the strength of evidence for having a functional interaction, a model selection step is performed for each pair of genes. We compute signal-to-noise ratios (SNRs), which are posterior odds for edge 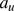 between a pair of genes 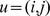 in favor of association to lack of association:
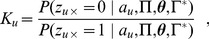
(1)where

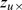
 is a latent variable indicating the affiliation of gene pair 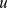 to mixture component 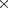 designating a lack of relationship;

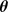
 denotes shape parameters of the three beta distributions;

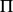
 denotes the set of mixture coefficients affiliated with different partition sets;

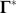
 is a matrix of hyperparameters of a Dirichlet prior with each row corresponding to a stratum and each column to a mixture component.

A cutoff score 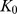 can be set to filter out non-significant edges, guided by the interpretation of Bayes factors by Harold Jeffreys [33]. The sign of each edge can be simply determined by comparing the posterior probabilities for it belonging to the mixture component representing positive and negative associations.

#### Searching for modules

We search for coherent functional modules in the inferred PAN by performing hierarchical clustering on functional association profiles, each of which is a vector of cosine similarities between one gene and all genes screened. The method compares functional profiles of genes instead of their individual functions, and it has been demonstrated to be a highly desirable measure to group genes with similar interaction patterns [9]. To assess the uncertainty of the clustering analysis, we computed a *p*-value for each cluster using multiscale bootstrap resampling details in section *Assessing the significance of cluster analysis* of Methods [18]. The clusters derived from hierarchical clustering are projected onto the inferred posterior association network to generate functional modules. Top significant modules enriched for significant functional interactions are selected according to four module filtering steps (Figure S1, details in section *Module filtering procedures* of Methods).

More details for the above procedures can be found in the Methods section.

### Simulation studies

In this section, we demonstrate the effectiveness of *PAN* by simulation studies on *in silico* data generated from multivariate normal distributions (details in section *Simulation settings* of Methods). We first assess effects of replicate sample size and network modularity strength on the performance of the global beta-mixture model. For the extended model, we test whether or not the performance can be improved by integrating prior information.

#### Evaluating the effect of replicate number and interaction strength

The performance of *PAN* can potentially be affected by (i) small replicate size and (ii) low degree of interaction strength in the network. A quantitative assessment of the impact of sample size is particularly important to help guide the experimental design to achieve the most cost-efficient solution.

**Figure 2:**
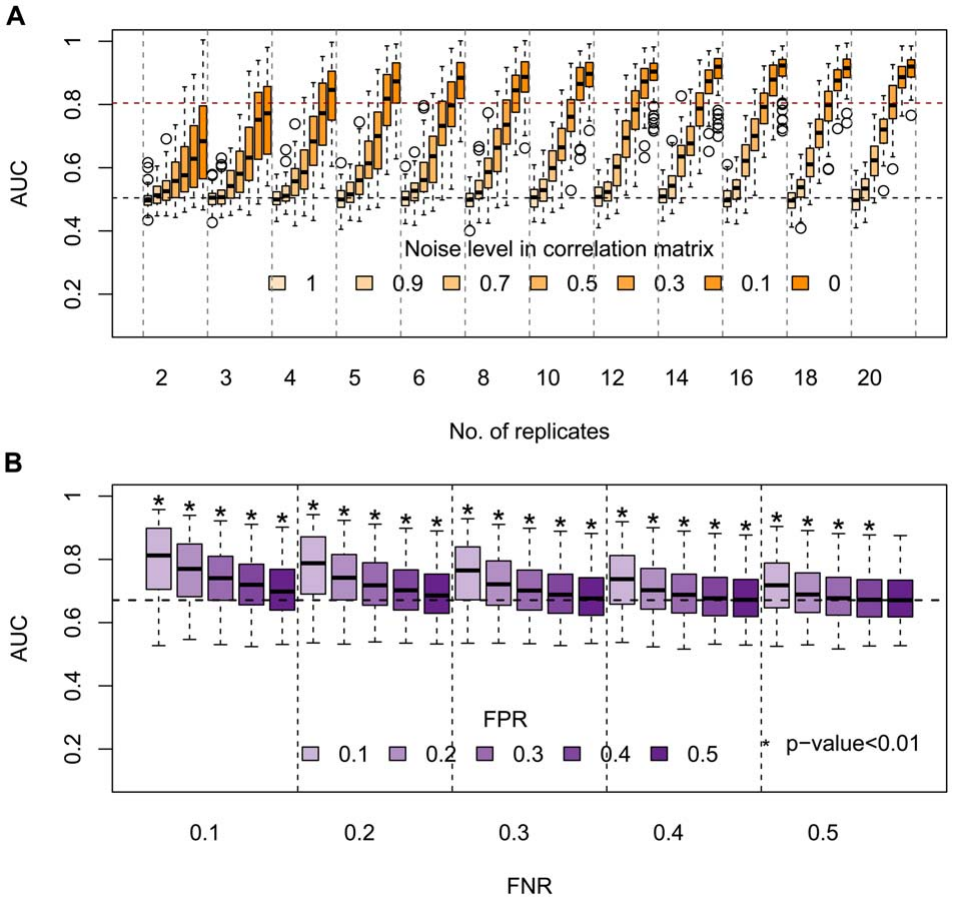
Results of simulation studies. (A) Simulation on the effect of replicate sample size and interaction strength. The black and red dashed lines indicate the base line (AUC = 0.5) and a high prediction performance (AUC = 0.8), respectively. The performance of *PAN*, as measured by AUC, increases along with the number of replicates and interaction strength. (B) Simulation on the effect of prior incorporation. The prediction accuracy (AUC = 0.65) of the global model is marked by the black dashed line. The performance is improved gradually by bringing in more prior information about functional interactions, but reduced by adding more noise in the prior.

In our simulations, we model replicate number by the sample size of a multivariate normal distribution and interaction strength by Pearson correlation coefficient. Considering 100 genes in total, we set two modules (with 30 genes for each) with positive internal interactions and negative external interactions to each other. We enumerated replicate size (from 2 to 20) and varied interaction strength by introducing random noise (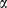 from 0 to 1) to the correlation matrix, which is used for data generation (details in *Simulation settings* of Methods). For each parameter setting we generated 100 random artificial screening data according to our simulation protocol. The global beta-mixture model (details in Methods) was applied to fit simulated data, and posterior odds were computed for each pair of genes. For each simulation, an AUC (area under the curve) score was computed by comparing gold-standards and predicted functional interactions by setting different cutoffs on the posterior odds. As expected, in general *PAN* performed better as the replication sample size increases and interaction strength increases (Figure 2(A)).

The simulation results suggest that our approach tends to identify those modules that are highly enriched for functional interactions. Increasing the number of replicates can help promote the prediction accuracy for modules with weaker interaction strength. When genes are completely randomly associated (100% random noise in the correlation matrix), as expected, *PAN* has a baseline performance (AUC = 0.5). For our two applications to real biological data, the replicate sizes (8 and 15, respectively) are sufficient for *PAN* to identify modules that are highly functionally connected. Nonetheless, the simulation results highlights the importance of incorporating additional knowledge to predict modules with an underestimated interaction strength reported by gene perturbation data (e.g. 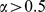).

#### Incorporating prior information significantly promotes model performance

In this simulation, we demonstrate that *PAN*'s prediction power can be greatly improved using the extended beta-mixture model, where complementary data is integrated. Such data can be often available in curated database of high quality (e.g. protein-protein interaction databases). False negative interactions are in general difficult to control and in order to minimize false positives ideal prior information should be from highly specialized or carefully filtered databases. For example, in our second application, we extracted protein-protein interactions from the PINdb (Database of Nuclear Protein Complexes [34]) and not other databases such as HPRD (Human Protein Reference Database [35]) or BioGRID [36], because we aimed at focussing on chromatin factors within the nucleus.

Taking one parameter setting (8 replicates, 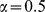) in the last simulations as an example, we stratify positive gold-standards (true edges) from negative ones (non-edges), and randomly flip 10%–50% edges and non-edges as false negatives and false positives. Compared with the baseline performance (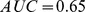) of the global model, the extended model resulted in significantly higher AUC scores as indicated by *p*-values computed from paired two sample t-tests except one extreme case (false positive rate or 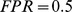, false negative rate or 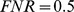) (Figure 2(B)). Interestingly, the extended model is more sensitive to false positives than false negatives. With a high false positive rate (e.g. 50%), the improvement in performance is not obvious; given a very low FPR (10%), however, the prediction power increases from 70% (FNR = 50%) up to about 80% (FNR = 10%). This suggests that with a careful control of FPR, the extended model has a great potential to increase the performance even with a low-level prior knowledge.

### Prioritizing a potential therapeutic network for Ewing's sarcoma

Having established our computational framework, we first demonstrate its general applicability on biological data sets that are publicly available. In this case study, we use RNAi phenotyping screens across multiple cell lines to infer functional modules of kinases that are critical for growth and proliferation of Ewing's sarcoma. We demonstrate that our model can make efficient use of single gene perturbation data to predict robust functional interactions.

#### A kinase screen in Ewing's sarcoma

The data used in this case study is a matrix (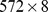) of Z-scores from high throughput RNAi screens run in duplicates targeting 572 human kinases in four Ewing's sarcoma cell lines: TC-32, TC-71, SK-ES-1 and RD-ES [4]. In these phenotyping screens, viability was assessed using a luminescence-based cell to quantify each gene's function in cancer cell growth and proliferation. The screening data was corrected for plate row variations and normalized using Z-score method as described in [4]. Compared to other RNAi screens in normal human fibroblast cell line, the four Ewing's sarcoma cell lines exhibited significant similarities, suggesting robust and consistent functional interactions among perturbed genes across cell lines [4].

#### Model assessment of *PANs*

**Figure 3:**
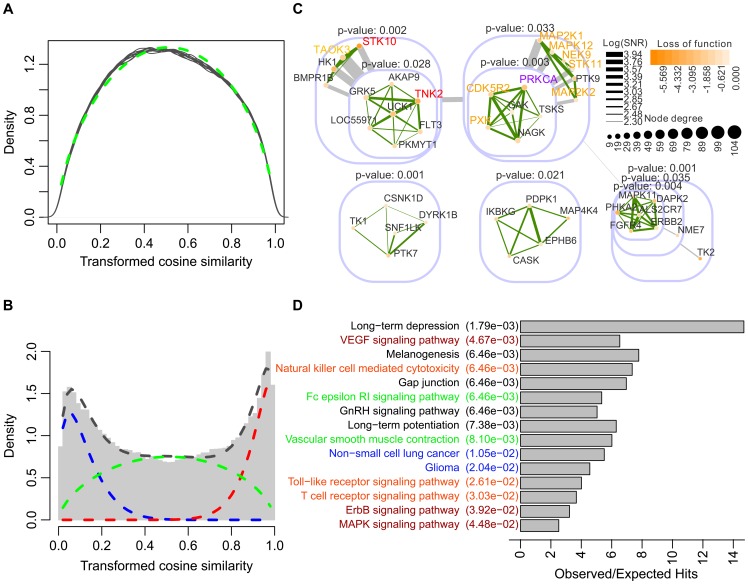
Application to Ewing's sarcoma. (A) Fitting a beta distribution to permuted screens. The transformed cosine similarity density curves of the permuted data are colored in grey. The fitted beta distribution is plotted as a dashed green curve. (B) Fitting a beta-mixture distribution to screening data. The transformed cosine similarities of the real screening data is shown in the grey histogram. Fitted beta distributions representing the 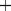, 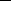 and 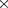 mixture component are plotted as red, blue and green dashed curves, respectively. The black dashed curve denotes the fitted mixed distribution. (C) Predicted significant modules. The significant modules predicted by *PAN* are presented in a nested structure. Each module is illustrated by a rounded rectangle including sub-modules and/or individual genes. The *p*-value (on the top of each module) computed by *pvclust* indicates the stability of genes being clustered together. *PRCKA* (the gene colored in purple) is known to be a kinase target for human sarcomas, and an inhibitor PKC412 targeting *PRCKA* has already been tested in the clinic. *STK10* and *TNK2* (colored in red) in the upper left module have been identified as potential therapeutic targets. Another eight genes (colored in yellow) in the upper left and right modules were also highly associated with apoptosis of Ewing's sarcoma. (D) Significantly overrepresented KEGG pathways. Hypergeometric tests were performed to evaluate overrepresentation of genes included in the upper right module in human KEGG pathways. Top significant pathways (*p*-value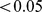) are ranked by *p*-value increasingly, and their corresponding ratios of the number of observed hits to expected hits are illustrated by a bar plot. Most of these significant pathways are related to cell proliferation (colored in red), smooth muscle contraction (colored in green), immune system response (colored in orange) and cancer (colored in blue).

To predict the functional interactions between genes, the proposed beta-mixture model was applied to quantify the significance of their associations, which are measured by cosine similarities computed from the Z-score matrix. We first permuted the Z-score matrix 20 times, computing cosine similarities and fitting a null distribution by maximum likelihood estimation using the function *fitdistr* of R package *MASS* (Figure 3(A)) [37]. The median values of the 20 fitted parameters were selected to fix the 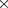 component representing lack of association in the mixture model. It should be noted that during the permutations, we kept the replication structure for each cell line. Our permutation strategy resulted in flat null distributions (Figure 3(A)), which yielded conservative estimation of the statistical significance of functional interactions in the following step.

Having fixed the parameters for the 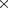 component, we performed MAP inference with an uninformative prior (uniform Dirichlet priors) to estimate the other parameters of the global mixture model using the EM algorithm introduced in Methods (fitting results shown in Figure 3(B)). Comparing the original histogram of cosine similarities, the fitted three beta distributions and the mixture of them, we found that the distribution of cosine similarities is successfully partitioned to three components capturing the population of signal (positive or negative association) and noise (lack of association). The posterior probabilities for each association belonging to different populations in the mixture model were computed subsequently for inference of the functional network.

### Identified modules are enriched for confirmed and potential therapeutic targets for Ewing's sarcoma

Having fitted the global mixture model to data successfully, we inferred a network of functional interactions between kinases based on the proposed edge inference approach. Setting the cutoff SNR score at 10, which is interpreted as a ‘strong’ evidence in Bayesian inference [33], we filtered out non-significant edges and obtained a very sparse network with 572 genes, only 5213 positive and 282 negative edges (3.36% of all gene pairs).

Hierarchical clustering with multiscale bootstrap resampling was conducted subsequently using the R package *pvclust* [18]. With 10000 times' resampling, we obtained 65 significant (*p*-value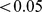) clusters with more than four genes. Of all these significant clusters, 30 clusters are enriched for functional interactions (module density 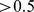). These clusters are superimposed to predicted posterior association networks to build functional modules (Figure S2). Here we focused on nine modules with genes that are associated with prohibition of cancer cell growth. These modules can be further collapsed to five ‘root’ modules and represented in a nested layout (using bioconductor package *RedeR* [38]) for better illustration of their relationships (Figure 3(C)).

The first module (upper left in Figure 3(C)) includes *STK10*, *TNK2* and *TAOK3*, which were identified to be significant across all four Ewing's sarcoma cell lines [4]. In particular, the roles of *STK10* and *TNK2* in inhibiting proliferation and inducing apoptosis upon knocking-down were confirmed by further RNAi using independent siRNAs, real-time kinetic analysis as well as image based analysis of annexin V staining [4]. Compared to the first module, the second module (upper right in Figure 3(C)) is even more interesting because most of genes in this module are important for proliferation of Ewing's sarcoma cells. *CDK5R2*, *NEK9*, *PRKCA*, *PXK* and *STK11* have significant effects on growth of cancer cells in all cancer cell lines [4]. Among these genes, *CDK5R2* seems to be worth exploring as a multi-CDK inhibitor that potentially targets *CDK5R2* has been studied clinically [39]. Moreover, MAP/MEK family kinases–*MAPK12*, *MAP2K1* and *MAP2K2* were also identified to be promising targets for pharmacological intervention in ES [40]. Even more strikingly, *PRKCA*, against which an inhibitor has already been tested extensively in the clinic, is also found in the second module [41].

### Pathways analysis reveals important roles of identified module in proliferation of Ewing's sarcoma cells

Previous RNAi screening studies such as [4] were dedicated to discovering single genes that are pivotal for inhibiting Ewing's sarcoma. In our predictions, genes in the module are densely connected with highly siginificant functional interactions, indicating possible genetic interactions may exist among them. If the hypothesis is true, these genes may be involved in the same biological processes. Focusing on genes in the second module, we further searched for kinase pathways in which they are enriched. Hypergeometric tests were performed on all genes in this module to test their overrepresentation in KEGG pathways using R package *HTSanalyzeR* [42]. In total, we identified 15 significant KEGG pathways (Benjamini-Hochberg adjusted *p*-value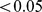) with 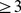 observed hits.

Among the top significant pathways (Figure 3(D)), VEGF, ErbB, and MAPK pathways are known to be critical for cell growth and proliferation. Many drugs have been designed to target the VEGF pathway using different strategies such as reducing VEGF expression by siRNAs and inhibiting VEGF receptor by antibodies [43]. The MAPK pathway was also identified to be a promising target for pharmacological intervention in Ewing's sarcoma [40]. Interestingly, we also identified Fc epsilon RI signalling pathway and Vascular smooth muscle contraction. It is known that a viable vascular supply is critical for Ewing's sarcoma tumors to grow, and in recently years, there is an increasing interest in inhibiting tumor vessel formation to treat Ewing's sarcoma. Many drugs have been designed under this strategy and have been evaluated in preclinical studies [43], [44].

Similar pathway analyses were also performed on the other four modules separately, but none of them are significantly overrepresented in any KEGG pathway. Taking all together, the second module is highly enriched for clinically confirmed and potential therapeutic targets, and associated with signalling pathways that are crucial for growth and proliferation of Ewing's sarcoma, demonstrating the prediction power of *PAN*.

### Predicting and confirming a genetic interaction network controlling stem cell fate

Having demonstrated its applicability, we applied the proposed computational framework to study self-renewal of epidermal stem cells using RNA interference screening data for 332 known and predicted chromatin modifiers. We predicted a highly significant module enriched for functional interactions, and confirmed their dense genetic interactions using combinatorial gene perturbation. Further experimental follow-up suggests that their genetic interactions may involve transcriptional cross regulations.

#### Data preprocessing

RNAi screening data were obtained for 332 chromatin factors under five conditions: vehicle, AG1478, BMP2/7, AG1478+BMP2/7 and serum stimulation in triplicates. In detail, siRNAs targeting these genes were placed in four 96-well plates, each of which includes two independent siRNAs targeting controls. For each well in each plate, the endogenous levels of transglutaminase I (TG1) protein and DRAQ5 signal were screened to measure differentiation per cell. TG1 is the key enzyme that mediates the assembly of the epidermal cornified envelope and is a marker of differentiated cells, while DRAQ5 signal is used to measure all cells. More details about the siRNA screening experiment can be found in our accompanying paper [5].

To correct for plate-to-plate variability, the raw screening measurement 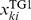 for 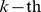 well in plate 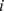 was normalized to DRAQ5 signal 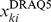 within the plate:
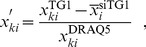
(2)where 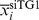 denotes the mean of control signals in plate 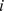. Z-scores were subsequently computed from the normalized data:
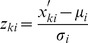
(3)where 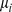 and 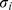 are the mean and standard deviation of all measurements within the 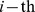 plate. After the above preprocessing steps, we obtained a 332 (genes) 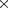
15 (3 replicates in 5 conditions) matrix of Z-scores.

#### Model assessment of *PANs*

**Figure 4:**
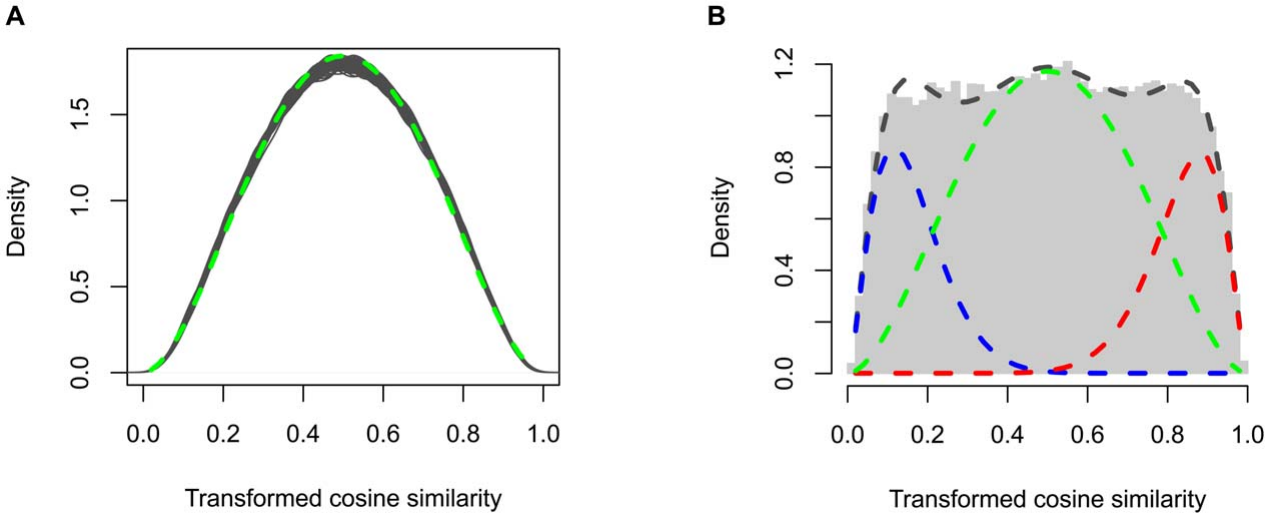
Fitting results of the global beta-mixture model. (A) Fitting a beta distribution to functional associations computed from permuted screening data. For each one of the total 100 permuted datasets, association densities were computed and a beta distribution was fitted. Each fitted distribution is plotted as a grey curve. The median scores of the two shape parameters of fitted beta distributions were selected to fix the 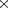 component (green dashed curve). (B) Fitting a global beta-mixture model to functional associations computed from the real screening data. The fitting is conducted based on the EM algorithm (details in Methods) with the shape parameters of the 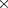 component fixed by fitting to permuted screening data. The histogram and the dashed curves show the real distribution of transformed association scores and the fitting result, respectively. Fitted distributions for positive, negative and lack of associations are illustrated by red, blue and green dashed curves, respectively.

Similar to the previous case study, we first fit the global mixture model to functional interaction profiles quantified by cosine similarities on the Z-score matrix. The fitting results of the null and mixture model are shown in Figure 4. The distribution of functional interactions is successfully partitioned to three mixture components. Using Equation (15), we computed posterior probabilities for each pair of genes having a positive, negative or no functional interaction.

### Predicted functional interactions are significantly enriched for protein-protein interactions

The matrix (54946 pairs of genes 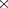
3 mixture components) of posterior probabilities were used to perform Gene set enrichment analysis (GSEA) [45] to test the hypothesis that proteins residing in the same complex are likely to be functionally connected. Different from conventional GSEA, here the ‘gene set’ is a set of gene pairs encoding protein complexes, whereas ‘phenotypes’ are posterior probabilities for all possible gene pairs belonging to each one of the three mixture components. We first build an adjacency matrix of protein-protein interactions (PPIs) from PINdb (version 2011-06-17), a high-quality literature-curated database of nuclear protein complexes [34]. In total, the matrix includes 9226 PPIs between 966 proteins. Mapping the 332 chromatin factors to the PPI matrix, we obtained 418 ‘gold standard’ interactions, which were used as our ‘gene set’ for the enrichment analysis.

**Figure 5:**
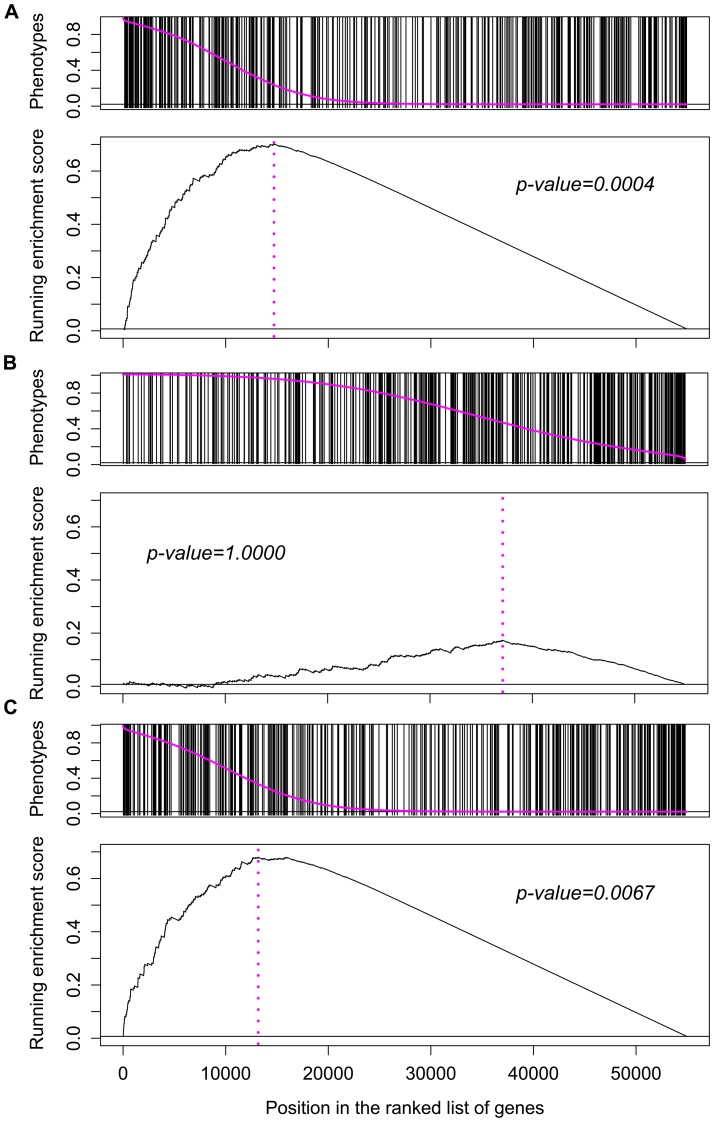
Enrichment of functional interactions for protein-protein interactions. (A), (B) and (C) correspond to enrichment analysis of protein-protein interactions (PPIs) in the posterior probabilities for associations belonging to the 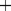, 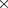 and 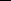 component, respectively. In each one of the three figures, the upper panel shows ranked phenotypes by a pink curve and the positions of PPIs in the ranked phenotypes, while the lower panel illustrates the running sum scores of GSEA (Gene Set Enrichment Analysis) random walks [45].

We performed GSEA for each mixture component using R package *HTSanalyzeR* [42] with permutation tests (10,000 permutations) to estimate enrichment significance. As expected, we obtained highly significant enrichment of PPIs in the 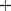 and 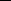 components (*p*-values are 0.0067 and 0.0004, respectively) but not in the 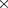 component (*p*-value = 1.0000) (Figure 5). This matches observations made in yeast where genetic interactions between complex components can be either aggravating or alleviating [9]. The enrichment results suggest a rationale for incorporating PPIs as *a priori* belief in predicting functional interactions.

### Improving functional interaction prediction by incorporating protein-protein interactions

As shown in the simulations, with complementary data our extended beta-mixture model can greatly improve prediction accuracy of functional interactions (Figure 2(B)). In real screening data, we also observe that there is indeed a significant enrichment of function interactions for protein-protein interactions (Figure 5). Here we take the advantage of such prior information to better predict functional connections using the extended model.

**Figure 6:**
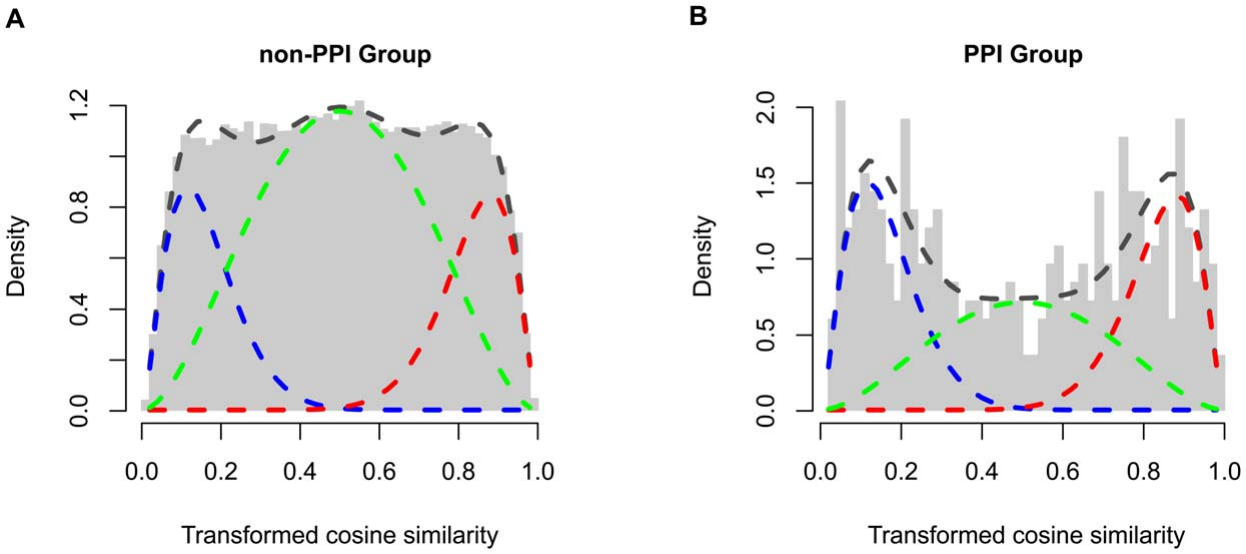
Fitting results of the extended beta-mixture model. The whole set of gene pairs are stratified to PPI(protein-protein interaction) group and non-PPI group. The extended beta-mixture model is fitted to functional associations, setting different prior probabilities (mixture coefficients) to these two groups. The fitting results for the PPI group is illustrated in (A), and the non-PPI group in (B). The histogram and the dashed curves show the real distribution of transformed association scores and the fitting result, respectively. Fitted distributions for positive, negative and lack of association are illustrated by red, blue and green dashed curves, respectively. The fitting results suggest that gene pairs in the PPI group have higher probability to be functionally connected than the non-PPI group.

Similarly, we first fit a null beta distributions to each of 100 perturbed data sets, and used the median values of the fitted parameters to fix the 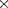 component in the mixture model. According to protein-protein interactions obtained from the PINdb database, we stratified the whole set of gene pairs to PPI group and non-PPI group. During the fitting to the extended model using the EM algorithm (details in Methods), different prior probabilities (mixture coefficients) for the three mixture components were used for these two groups. As expected, the fitted mixture coefficients of the 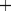 and 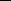 components for the PPI group (30.4% and 30.9%) are significantly higher than the non-PPI group (18.2% and 17.9%). The fitting results suggest that gene pairs in the PPI group are much more likely to be positively or negatively associated (Figure 6).

### Predicted posterior association network and functional modules

Based on the fitting results of the extended mixture model, we next inferred a network of functional interactions between the chromatin factors. We weighted the edges using signal-to-noise ratios (SNRs), which are essentially posterior odds of gene pairs in favor of signal (association) to noise (lack of association). The sign of each edge was determined by comparing the posterior probabilities belonging to the positive and negative association components. Setting a cutoff SNR score at 10, we obtained a sparse network with 165 genes, only 848 positive and 878 negative edges (12.8% of all gene pairs).

To assess the uncertainty of the clustering analysis, we computed a *p*-value for each cluster using multiscale bootstrap resampling using *pvclust* [18]. With 10000 times' resampling we obtained 39 significant clusters (*p*-value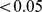) including 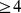 genes. Mapping these gene clusters to the inferred functional network, we identified 13 tightly connected modules (density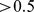). Similar to the application to Ewing's sarcoma, we can visualize the modules in a nested structure (Figure S3(A)). Using the prior PPI network from the PINdb database, we applied the same module filtering strategy and found no module enriched for known PPIs due to sparsity of PPIs (Table S1). Even when relaxing the cutoff on module density to 0, far fewer modules were found by PPI (10) (Figure S3(B)) than by *PAN* (22). Thus, although the PPI network correlates with our predicted functional interactions using *PAN* (as shown in Figure 5), using it alone is not informative as *PANs*.

**Figure 7:**
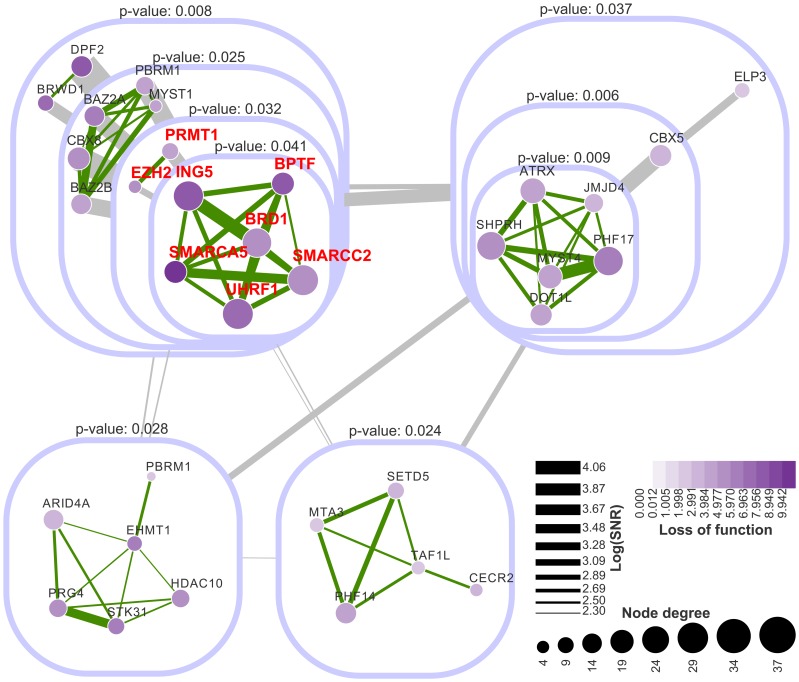
Top significant modules predicted by *PAN*. Nodes with purple colors represent positive perturbation effects. Node colors are scaled according to their averaged perturbation effects under the vehicle condition. Node sizes are scaled in proportion to their degrees. Edge widths are in proportion to log signal-to-noise ratios. Edges colored in green and grey represent positive interactions inside modules and summed interactions between modules, respectively. This figure illustrates top significant modules and their dense functional interactions. Genes colored in red were selected for further experimental investigation.

Of all modules predicted using *PAN*, we focused on nine modules with positive effects upon perturbation indicating that they are associated with self-renewal of epidermal stem cells (Figure 7). We found the module including *ING5*, *BRD1*, *BPTF*, *SMARCA5*, *UHRF1*, *EZH1*, *SMARCC2* and *PRMT1* of particular interest, as it functionally connects two factors known to regulate keratinocyte self-renewal (*EZH2* and *UHRF1*) with factors that have not been implicated previously(*ING5*, *SMARCA5* and *BPTF*). Thus, we focused on genes in this module and performed experimental follow-up to further investigate the potential molecular basis for these functional interactions.

### Experimental validations

**Figure 8:**
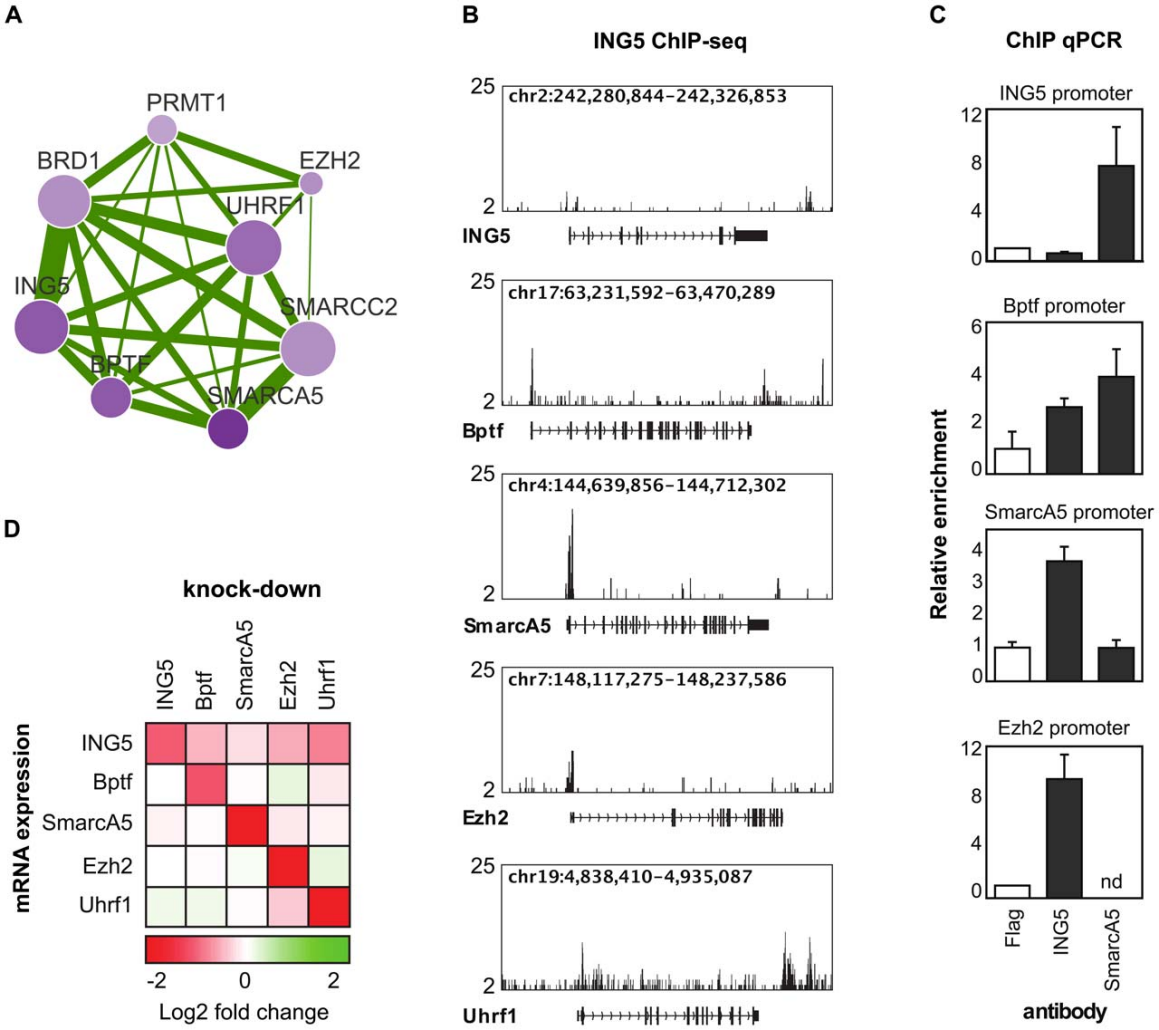
Validating predicted functional module. (A) The predicted functional module examined by further experiments. Figure legends are the same as Figure 7. (B) Genome browser tracks of *ING5* ChIP-seq signals on the loci of *ING5*, *BPTF*, *SMARCA5*, *EZH2* and *UHRF1*. These figures show a strong signal of *ING5* binding to *BPTF*, *SMARCA5* and *EZH2*. (C) ChIP-qPCR experiments of *ING5* and *SMARCA5* binding to *ING5*, *BPTF*, *SMARCA5* and *EZH2*. These figures further confirm the occupancy of *ING5* on the promoters of *BPTF*, *SMARCA5* and *EZH2*, and suggest that *SMARCA5* binds to *ING5* and *SMARCA5*. ‘nd’ in the last panel means not determined. (D) RT-qPCR examining mRNA expression changes (log fold change) of *ING5*, *BPTF*, *SMARCA5*, *EZH2* and *UHRF1* after perturbing each one of them. This figure indicates feedback regulations of *BPTF*, *SMARCA5*, *EZH2* and *UHRF1* on *ING5*.

The dense functional connections between *ING5*, *BRD1*, *BPTF*, *SMARCA5*, *UHRF1*, *EZH2*, *SMARCC2* and *PRMT1* suggest potential enriched genetic interactions (Figure 8(A)). We examined the synthetic genetic interactions between *ING5*, *BPTF*, *SMARCA5*, *EZH2* and *UHRF1* by combinatorial knock-down experiments using two independent sets of siRNAs. As expected, we observed significant combinatorial effects in eight out of ten gene pairs validating the prediction power of *PAN* (details in our accompanying paper [5]).

To understand the basis of their genetic interactions, we further looked for possible transcriptional regulation among them. Chromatin immunoprecipitation coupled with massively parallel sequencing (ChIP-seq) analysis was conducted for *ING5* to check its binding events on a genome-wide scale. Significant peaks were observed in the promoter regions of *BPTF*, *SMARCA5* and *EZH2* but not *ING5* itself or *UHRF1*, suggesting that *ING5* is a co-regulator of these three genes (Figure 8(B)). This conclusion is also confirmed by ChIP-qPCR examination of *ING5* binding to the other four genes (Figure 8(C)).

Interestingly, in our ChIP-qPCR analysing *SMARCA5* binding events, we observed *SMARCA5* occupying the promoter of *ING5* and *BPTF* (Figure 8(C)), indicating a feedback transcriptional regulation between *ING5* and *SMARCA5*. More feedback regulation was also found between *BPTF*, *EZH2* and *UHRF1* on *ING5*. In further RT-qPCR experiments, significant changes in mRNA expression of *ING5* were observed when perturbing *BPTF*, *SMARCA5*, *EZH2* and *UHRF1* (Figure 8(D)). Taking all our experimental results together, the basis of the genetic interactions between these five genes involves transcriptional cross regulations.

## Discussion

Recent years have seen an increasing interest in using massive combinatorial perturbations to study genetic interactions systematically. This approach has only been applied to model organisms such as yeast and bacteria on a large scale due to its limited scalability on metazoans. In this paper, we reported a scalable and affordable strategy to predict functional interactions from single gene perturbation screens. As demonstrated in our two applications, *PAN* can not only be applied to custom data sets, but also be implemented on public databases (e.g. GenomeRNAi [28]) including phenotyping screens of single gene perturbations or chemical compound treatments obtained from different cell lines or various biochemical conditions. Our approach also has the potential to explore functional interactions from new types of phenotyping screens such as multiparametric high-content imaging data [2], [3].

As shown in our second case study, protein-protein interactions are found to be significantly enriched for functional interactions. Such prior information is informative but poses big challenges to conventional parametric or permutation-based nonparametric hypothesis tests. *PAN* naturally addresses the challenge by employing a stratified beta mixture model, which allows different prior probabilities for gene subpopulations with different levels of modularity. Our simulation study demonstrated that the extended model can greatly improve the prediction power for networks with underestimated modularities reported by gene perturbations. Nevertheless, signal-to-noise ratios derived from our global beta mixture model and *p*-values derived from permutation-based hypothesis testing can be mapped to each other for convenience (Table S2).

To show the general applicability to real biological data, we applied *PAN* to a public dataset of Ewing's sarcoma (ES) and prioritized a potential therapeutic network that is highly enriched for druggable genes and associated with pathways that are known to be critical for growth and survival of ES cells. Using our own custom generated RNAi screens of chromatin factors under five different biochemical treatments, *PAN* identified a highly enriched module controlling human epidermal stem cell fate. The predicted functional interactions between selected five genes in the module were further confirmed by combinatorial RNAi experiments. ChIP-seq, ChIP-qPCR and RT-PCR experiments revealed transcriptional cross regulation among these genes, which may explain their genetic interactions.

In our two applications, only a handful of top significant modules are obtained because: a) a stringent SNR cutoff was deliberately chosen to select highly significant functional interactions, and b) a few filtering steps are involved to select modules enriched for significant interactions (Figure S1). Relaxing either SNR cutoff during *PAN* inference or module filtering constraints can increase the number of modules (Table S3). For example, making a SNR cutoff at 10 and considering both positive and negative loss-of-function, we obtained 13 (Figure S3(B)) and 30 modules (Figure S2) in the application to epidermal stem cells and Ewing's sarcoma, respectively. Many modules in Figure S2 and Figure S3(B) that are not shown in Figure 3 or Figure 7 may also be of interest to other researchers, although they are out of the scope of this paper. For example, dense functional interactions were detected between *CHD4*, *BRDT*, *BRD4* and *PHF1* (in Figure S3(A)), indicating possible genetic interactions among these genes regulating epidermal differentiation.

Although not found in our applications, it could happen in principle that no phenotypic change is observed upon single gene perturbation. These extreme cases could be explained when two genes in two distinct but combinatorial pathways fully compensate each other function. The functional associations between these genes have much higher chance to belong to the 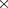 subpopulation in our mixture model, and will probably be false negatives. This is a challenge for not only our approach, but also the other potential computational methods as well as biologists. One solution would be to test these genes (a small number expected) together with genes in the modules predicted by *PAN* using combinatorial perturbation.

## Methods

### Cosine similarity

Cosine similarity is a measure of similarity by computing the cosine of the angle between two vectors. Let 
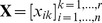
 be a matrix of measured phenotypes, in which 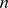 and 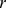 denote the number of genes and replicates in the experiment, respectively. The cosine similarity here between gene 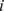 and 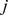 is their normalized dot product, namely:
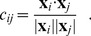
(4)A cosine similarity ranges from −1 (exactly opposite) to 1 (exactly the same) with 0 indicating a lack of relationship. The biological meaning for a positive or negative cosine similarity is that two genes are positively or negatively regulated, affected or functionally related, depending on the type of phenotype measured.

### The global beta-mixture model

Finite mixture models have been used to identify co-expressed genes from gene expression data [46]. An efficient methodology proposed by Ji *et al.* [47] models densities of correlation coefficients of gene expression levels by a mixture of a finite number of beta distributions. Here, we apply this approach to model associations of phenotypic readouts and extend it to integrate complementary data sources.

For simplicity, we denote the set of association scores (e.g. cosine similarities) as 
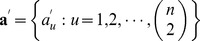
. To fit the range of beta-distributions, we use linearly transformed scores 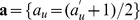.

We assume that 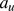 follow a mixture of three beta distributions, namely:
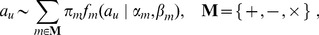
(5)where 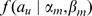 is a beta density function with 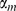 and 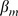 as shape parameters.

Let 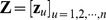 be a matrix of hidden data, where 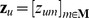 is a vector of latent indicator variables for gene pair 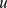, in which:
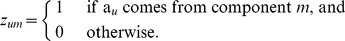
(6)

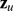
 is independent and identically distributed according to an three-category multinomial distribution with probabilities 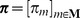. The likelihood of the sets of parameter 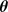 and 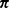 given the complete data 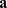 and 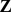 is:
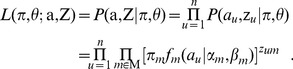
(7)The logarithm of the above likelihood is:
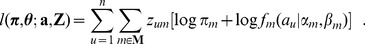
(8)Based on the log-likelihood function, Ji et al. proposed an Expectation-Maximization (EM) algorithm [32] to estimate parameters [47].

### The extended beta-mixture model

We demonstrated in our application to epidermal stem cells that gene pairs with evidences of protein-protein interactions in the nucleus tend to have higher functional associations. However, such prior information is ignored in the above global mixture model, which treats every association equally multinomially distributed with the same parameters. Inspired by the stratified Gaussian mixture model proposed by Pan *et al.* for clustering of microarray data [48], we extend to a stratified beta mixture model to incorporate potential prior information.

#### Stratifying functional interactions

The full set of associations 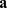 is partitioned to disjoint subsets 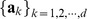 (e.g. subsets of associations with and without PPIs as discussed later in section). Consequently, the stratified probability density function becomes:
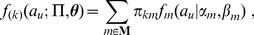
(9)in which 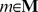 specifies the mixture component and 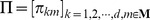 denotes the set of mixture coefficients affiliated with different partition sets. Correspondingly, we derive the extended log-likelihood:
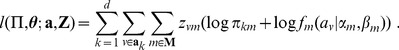
(10)


#### Bayesian regularization

To obtain smoother estimates of the parameters and guide the selection of model structures, we perform Bayesian regularization for the mixture model by introducing Dirichlet priors for the likelihood:
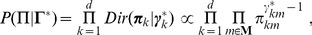
(11)where 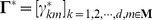 is a matrix of hyper-parameters for the dirichlet prior with each row corresponding to a stratum and each column to a mixture component. The posterior probability can be written as:
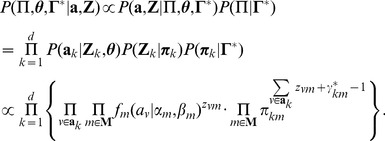
(12)


The corresponding log-posterior probability is:
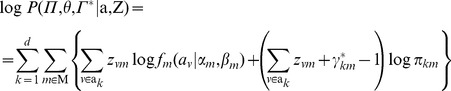
(13)


For a Dirichlet prior distribution 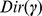, to specify the hyper-parameters we adopt the following decomposition:
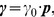
(14)where 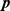 is a prior distribution normalized to 1 specifying the prior beliefs towards different mixture components and 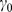 is a scale parameter specifying the strength of prior beliefs.

#### Posterior probability

Having estimated the paramters in the beta-mixture model, the posterior probability for association 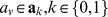 belonging to the 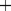, 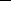 or 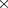 mixture component can be computed by:
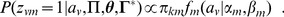
(15)


### Maximum *a posteriori* (MAP) inference

We propose to perform MAP estimation using a similar EM algorithm as Ji et al., which alternates between computing the expectation of the log-posterior probability based on the current estimates for the latent variables and maximizing the expected log-posterior:

- **E**xpectation**-step**: Given currently estimated parameters and latent variables, the expected value of the log-posterior probability is:
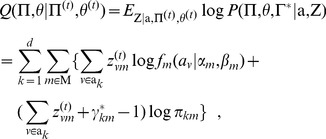
(16)where for association 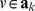:
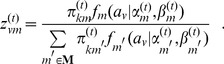
(17)
- **M**aximization**-step**: Update the estimates for parameter 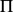 and 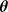 to optimize the expected value in Eq. (16). Derived from the partial derivatives of the Q function with respect to the mixture coefficients, the updating function is obtained as follows:
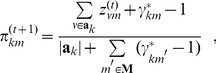
(18)where 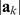 is the length of 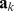. When 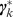 is uniformly distributed for 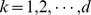, the MAP estimation degenerates to ML estimation.

Due to the difficulty to derive a closed-form expression to estimate the parameters of beta distributions, similar to Ji et al. [47] we use the ‘nlm’ function in R [49] to fit these parameters numerically.

In practice, our method differs from the global beta-mixture model proposed by Ji et al. in the following aspects:

- The global beta-mixture model proposed by Ji et al. has a challenge to determine the number of beta distributions using a model selection criterion (e.g. AIC, BIC or ICL-BIC). We deliberately apply a three-component beta-mixture model to fit association densities of perturbation screens under a very reasonable biological assumption as we discussed before.
- We fit a beta distribution to association scores computed from permuted screening data to fix the mixture component representing lack of association. This strategy can help avoid potential overfitting in the global model.
- Our extended stratified mixture model allows integration of prior knowledge such as protein-protein interactions.

### Simulation settings

The preprocessed phenotyping screens can be considered as samples drawn from multivariate normal distributions. Considering 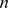 genes perturbed in an RNAi experiment, we paritition them to three groups 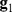, 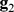 and 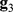 with the size of 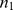, 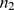 and 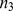. Genes in group 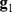 and 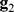 are associated with positive and negative perturbation effects, respectively. Genes in group 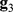 can have either positive or negative loss of functions. The following steps are involved to produce an artificial phenotyping screens.

**Figure 9:**
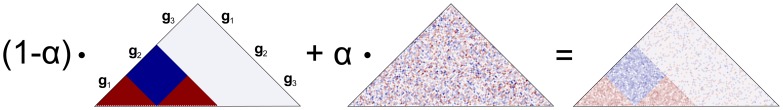
Correlation matrix simulation. In the ‘signal’ matrix (the left triangular matrix), 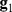, 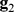 and 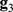 represent genes that have positive, negative and random perturbation effects, respectively. Matrix space colored in dark red and dark blue denotes positive and negative association, and white lack of association. These three spaces are filled in Pearson correlation coefficients of 1, −1 and 0, respectively. The ‘noise’ matrix (the middle triangular matrix) is a random correlation matrix generated using function ‘rcorr’ in R package *ggm*. The correlation matrix (the right triangular matrix) is then generated by a weighted sum of the ‘signal’ matrix and the ‘noise’ correlation matrix.

- **Set up a correlation matrix.** The correlation matrix is generated by a weighted sum of a ‘signal’ matrix 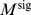 and a ‘noise’ correlation matrix 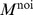 (Figure 9). In the ‘signal’ matrix, the entries within group 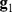 and 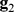 are set to 1, while the entries between the two groups are set to −1. All the other entries in the ‘signal’ matrix are set to 0. The ‘noise’ matrix is a random correlation matrix generated using function ‘rcorr’ in R package *ggm* (based the method in [50]). The weighted sum of the two matrices 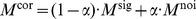, where 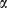 denotes the proportion of noise, is used in the following steps.
- **Generate random sample means and standard deviations.** To approximate the real data, we first compute sample means 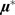 and standard deviations 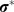 from the screening data set in our application to epidermal stem cells. For genes in 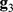, we draw sample means from 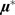 directly. For genes in 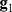 and 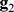, their corresponding sample means are randomly drawn from 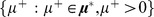 and 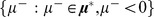, respectively. All sample standard deviations are drawn from 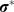 randomly.
- **Transform to covariance matrix.** The correlation matrix 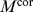 are transformed to covariance matrix 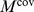 by multiplying diagonal entry 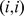 with variance 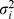 and non-diagonal entry 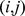 with product of standard deviations 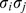.
- **Generate samples from covariance matrix.** Having obtained a covariance matrix, the artificial screens can be generated by drawing random samples with a given replicate size.

### Assessing the significance of cluster analysis

To evaluate the uncertainty of cluster analysis, a conventional approach is to perform ordinary bootstrap resampling of data [51]. Cluster analysis is then repeatedly applied to boostrap samples to obtain bootstrap replicates of the cluster dendrogram. A bootstrap probability can be calculated subsequently for each cluster simply by counting how frequent it appears in the bootstrap replicates. However, bootstrap probabilities are known to be biased due to comparing many dendrograms at the same time (detailed discussion in [52]). To reduce the bias, an approximately unbiased (AU) test was developed to calculate more accurate probabilities by *multiscale bootstrap resampling*, which means varying the sample size during resampling. In the AU test, an AU probability is calculated for each cluster by fitting a regression model to observed bootstrap probabilities (see [52] for an analytic description of the method). AU probabilities have been proved by an asymptotic theory to be less biased than conventional bootstrap probabilities [52], and have been widely used in many applications to assess cluster significance. The cluster *p*-values we used in the paper are defined as one minus AU probabilities. In the two applications, cluster *p*-values were computed using R package *pvclust* [18], varying the bootstrap sample size from 0.5 to 1.4 fold the real sample size of screening data. Clusters with *p*-values lower than 5% are strongly supported by the screening data and are selected to be significant modules.

### Module filtering procedures

Functional modules are generated by superimposing clusters, obtained from hierarchical clustering on functional profiles, onto inferred posterior associated networks. To select highly significant functional modules, we applied a few filtering procedures (Figure S1) including:

- Select significant modules that are strongly supported by data. The significance of clusters is quantified by *p*-values derived from hierarchical clustering with multiscale bootstrap resampling described in the previous section.
- Exclude extremely big or small modules. *PANs* aim at predicting interesting and experimentally testable hypotheses, thus modules that are extremely big or small are filtered out. In our two applications, modules with more than half of the total number of genes or less than five genes are excluded.
- Select modules that are densely functionally connected. Graph (or module) density, the ratio of predicted significant associations to all possible associations, is computed for each module to assess how densely genes are functionally connected.
- Select modules associated with specific function of interest. Identified functional modules could be dominated by genes associated with positive or negative loss-of-functions. This filtering step can be applied in many real applications to focus on a specific function of interest. For example, in the application to epidermal stem cells, modules associated with positive loss-of-function (increased differentiation upon perturbation) were selected because we are only interested in chromatin factors regulating self-renewal.

### Experimental methods

Chromatin immunoprecipitations were performed as described in our accompanying paper [5]. In short, primary human keratinocytes were grown on an irradiated J2-3T3 feeder layer. At 80% confluency feeders were removed, keratinocytes harvested by trypsinisation and crosslinked for 10 minutes with 1% formaldehyde. Nuclei were isolated by hypotonic lysis and DNA fragmented by sonication. Proteins were immunoprecipitated using anti-ING5 or SmarcA5 antibodies (both AbNova) overnight at 4 degrees Celsius and captured on 100 microliters of protein G coated magnetic beads (Invitrogen) followed by 5 washes in RIPA buffer. For quantitative PCR analysis, DNA was analysed using a SYBR green based method (Applied Biosystems). ING5 sequencing libraries were prepared as described in [53] and sequenced on an Illumina Genome Analyser II. Data was handled as described in [5]. For reverse transcription (RT)-qPCR, cDNA was generated using the Superscript Supermix for qPCR (Invitrogen) and subjected to SYBR green based quantitative PCR.

## Supporting Information

Figure S1 **Module filtering procedures.** The schematic figure illustrates the four procedures to filter modules in *PANs*.
(PDF)

Figure S2 **Predicted significant modules for Ewing's sarcoma.** This figure is a more complete version for Figure 3(C). It includes also modules that are associated with positive loss of function (increased cancer cell viability) upon perturbation. The legends are the same as Figure 3(C) except that genes with positive perturbation phenotypes are colored in purple.
(PDF)

Figure S3
**Predicted significant modules for epidermal stem cells.** (A) The figure is a more complete version for Figure 7. It includes also modules that are associated with negative loss of function (decreased differentiation) upon perturbation. The legends are the same as Figure 7 except that genes with negative perturbation phenotypes are colored in orange. (B) The figure represent modules filtered using only the prior protein-protein interaction network. Ten modules in four root modules are obtained when filtering by a very baseline cutoff (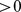). Using the same module density cutoff (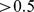) as *PAN*, however, no significant module is obtained. The two figures suggest that the prior PPI network alone is not as informative as *PAN* in identifying functional modules.(PDF)

Table S1 **NO. of modules obtained at each filtering step using PAN or PPI for epidermal stem cells.**
(DOC)

Table S2 **Mapping between** ***p*** **-values and signal-to-noise ratios.** (A) Mapping *p*-values to SNRs in the application to epidermal stem cells; (B) Mapping SNRs to *p*-values in the application to epidermal stem cells; (C) Mapping *p*-values to SNRs in the application to Ewing's sarcoma; (D) Mapping SNRs to *p*-values in the application to Ewing's sarcoma;
(DOC)

Table S3 **No. of modules obtained at each filtering step varying the SNR cutoff.** (A) and (B) are for the application to epidermal stem cells and Ewing's sarcoma, respectively.
(DOC)

## References

[1] Bakal C, Aach J, Church G, Perrimon N (2007). Quantitative morphological signatures define local signaling networks regulating cell morphology.. Science.

[2] Fuchs F, Pau G, Kranz D, Sklyar O, Budjan C (2010). Clustering phenotype populations by genome-wide RNAi and multiparametric imaging.. Mol Syst Biol.

[3] Green R, Kao H, Audhya A, Arur S, Mayers J (2011). A high-resolution C. elegans essential gene network based on phenotypic profiling of a complex tissue.. Cell.

[4] Arora S, Gonzales I, Hagelstrom R, Beaudry C, Choudhary A (2010). RNAi phenotype profiling of kinases identifies potential therapeutic targets in Ewing's sarcoma.. Mol Cancer.

[5] Mulder K, Wang X, Escriu C, Ito Y, Schwarz R (2012). Diverse epigenetic strategies interact to control epidermal differentiation.. Nat Cell Biol.

[6] Boutros M, Ahringer J (2008). The art and design of genetic screens: RNA interference.. Nat Rev Genet.

[7] Mani R, St Onge R, Hartman J, Giaever G, Roth F (2008). Defining genetic interaction.. Proc Natl Acad Sci U S A.

[8] Baryshnikova A, Costanzo M, Kim Y, Ding H, Koh J (2010). Quantitative analysis of fitness and genetic interactions in yeast on a genome scale.. Nat Methods.

[9] Costanzo M, Baryshnikova A, Bellay J, Kim Y, Spear E (2010). The genetic landscape of a cell.. Science.

[10] Battle A, Jonikas M, Walter P, Weissman J, Koller D (2010). Automated identification of pathways from quantitative genetic interaction data.. Mol Syst Biol.

[11] Farha M, Brown E (2010). Chemical probes of escherichia coli uncovered through chemical-chemical interaction profiling with compounds of known biological activity.. Chem Biol.

[12] Tong A, Lesage G, Bader G, Ding H, Xu H (2004). Global mapping of the yeast genetic interaction network.. Science.

[13] Collins S, Miller K, Maas N, Roguev A, Fillingham J (2007). Functional dissection of protein complexes involved in yeast chromosome biology using a genetic interaction map.. Nature.

[14] Horn T, Sandmann T, Fischer B, Axelsson E, Huber W (2011). Mapping of signaling networks through synthetic genetic interaction analysis by rnai.. Nat Methods.

[15] Echeverri C, Perrimon N (2006). High-throughput RNAi screening in cultured cells: a user's guide.. Nat Rev Genet.

[16] Echeverri C, Beachy P, Baum B, Boutros M, Buchholz F (2006). Minimizing the risk of reporting false positives in large-scale RNAi screens.. Nat Methods.

[17] Booker M, Samsonova A, Kwon Y, Flockhart I, Mohr S (2011). False negative rates in Drosophila cell-based RNAi screens: a case study.. BMC genomics.

[18] Suzuki R, Shimodaira H (2006). Pvclust: an R package for assessing the uncertainty in hierarchical clustering.. Bioinformatics.

[19] Wong S, Zhang L, Tong A, Li Z, Goldberg D (2004). Combining biological networks to predict genetic interactions.. Proc Natl Acad Sci U S A.

[20] Kelley R, Ideker T (2005). Systematic interpretation of genetic interactions using protein networks.. Nat Biotechnol.

[21] Le Meur N, Gentleman R (2008). Modeling synthetic lethality.. Genome Biol.

[22] Szappanos B, Kovcs K, Szamecz B, Honti F, Costanzo M (2011). An integrated approach to characterize genetic interaction networks in yeast metabolism.. Nat Genet.

[23] Lee I, Lehner B, Vavouri T, Shin J, Fraser A (2010). Predicting genetic modiffer loci using functional gene networks.. Genome Res.

[24] Zhong W, Sternberg P (2006). Genome-wide prediction of C. elegans genetic interactions.. Science.

[25] Qi Y, Suhail Y, Lin Y, Boeke J, Bader J (2008). Finding friends and enemies in an enemies-only network: a graph diffusion kernel for predicting novel genetic interactions and co-complex membership from yeast genetic interactions.. Genome Res.

[26] Flint J, Mackay T (2009). Genetic architecture of quantitative traits in mice, ies, and humans.. Genome Res.

[27] Rzhetsky A, Zheng T, Weinreb C (2006). Self-correcting maps of molecular pathways.. PLoS one.

[28] Gilsdorf M, Horn T, Arziman Z, Pelz O, Kiner E (2010). GenomeRNAi: a database for cell-based RNAi phenotypes. 2009 update.. Nucleic Acids Res.

[29] Eisen M, Spellman P, Brown P, Botstein D (1998). Cluster analysis and display of genome-wide expression patterns.. Proc Natl Acad Sci U S A.

[30] Dadgostar H, Zarnegar B, Hoffmann A, Qin X, Truong U (2002). Cooperation of multiple signaling pathways in CD40-regulated gene expression in B lymphocytes.. Proc Natl Acad Sci U S A.

[31] de Hoon M, Imoto S, Miyano S (2002). A comparison of clustering techniques for gene expression data.. http://bonsai.hgc.jp/mdehoon/publications/ismb2002.pdf.

[32] Dempster A, Laird N, Rubin D (1977). Maximum likelihood from incomplete data via the EM algorithm.. J Roy Stat Soc B Met.

[33] Jeffeys H (1998). Theory of probability. 3rd edition.

[34] Luc P, Tempst P (2004). PINdb: a database of nuclear protein complexes from human and yeast.. Bioinformatics.

[35] Prasad T, Goel R, Kandasamy K, Keerthikumar S, Kumar S (2009). Human protein reference database2009 update.. Nucleic Acids Res.

[36] Stark C, Breitkreutz B, Chatr-Aryamontri A, Boucher L, Oughtred R (2011). The biogrid interaction database: 2011 update.. Nucleic Acids Res.

[37] Venables WN, Ripley BD (2002). Modern Applied Statistics with S 4th edition.

[38] Castro M, Wang X, Fletcher M, Meyer K, Markowetz F (2012). RedeR: R/Bioconductor package for representing modular structures, nested networks and multiple levels of hierarchical associations.. Genome Biol.

[39] Tibes R, Jimeno A, Von Hoff D, Walker R, Pacciarini M (2008). Phase I dose escalation study of the oral multi-CDK inhibitor PHA-848125.. J Clin Oncol.

[40] Benini S, Manara M, Cerisano V, Perdichizzi S, Strammiello R (2004). Contribution of MEK/MAPK and PI3-K signaling pathway to the malignant behavior of Ewing's sarcoma cells: Therapeutic prospects.. Int J Cancer.

[41] Kawamoto T, Akisue T, Kishimoto K, Hara H, Imabori M (2008). Inhibition of PKC*α* Activation in Human Bone and Soft Tissue Sarcoma Cells by the Selective PKC Inhibitor PKC412.. Anticancer Res.

[42] Wang X, Terfve C, Rose J, Markowetz F (2011). HTSanalyzeR: an R/Bioconductor package for integrated network analysis of high-throughput screens.. Bioinformatics.

[43] DuBois S, Marina N, Glade-Bender J (2010). Angiogenesis and vascular targeting in Ewing sarcoma.. Cancer.

[44] Schadler K, Zweidler-McKay P, Guan H, Kleinerman E (2010). Delta-like ligand 4 plays a critical role in pericyte/vascular smooth muscle cell formation during vasculogenesis and tumor vessel expansion in Ewing's sarcoma.. Clin Cancer Res.

[45] Subramanian A, Tamayo P, Mootha V, Mukherjee S, Ebert B (2005). Gene set enrichment analysis: a knowledge-based approach for interpreting genome-wide expression profiles.. Proc Natl Acad Sci U S A.

[46] McLachlan G, Peel D (2000). Finite mixture models.

[47] Ji Y, Wu C, Liu P, Wang J, Coombes K (2005). Applications of beta-mixture models in bioinformatics.. Bioinformatics.

[48] Pan W (2006). Incorporating gene functions as priors in model-based clustering of microarray gene expression data.. Bioinformatics.

[49] R Development Core Team (2011). R: A Language and Environment for Statistical Computing, version 14.

[50] Marsaglia G, Olkin I (1984). Generating correlation matrices.. SIAM J Sci Stat Comp.

[51] Felsenstein J (1985). Confidence limits on phylogenies: An approach using the bootstrap.. Evolution.

[52] Shimodaira H (2002). An approximately unbiased test of phylogenetic tree selection.. Syst Biol.

[53] Schmidt D, Wilson M, Spyrou C, Brown G, Hadfield J (2009). ChIP-seq: using high-throughput sequencing to discover protein-DNA interactions.. Methods.

