# Supplementary material for: Posterior Association Networks and Functional Modules Inferred from Rich Phenotypes of Gene Perturbations
Source: PLoS Comput Biol. 2012 Jun 28;8(6):e1002566. doi: 10.1371/journal.pcbi.1002566 (PMC3386165; doi:10.1371/journal.pcbi.1002566)
Supplement: Figure S1 — Module filtering procedures. The schematic figure illustrates the four procedures to filter modules in PANs. (PDF) [file pcbi.1002566.s001.pdf]

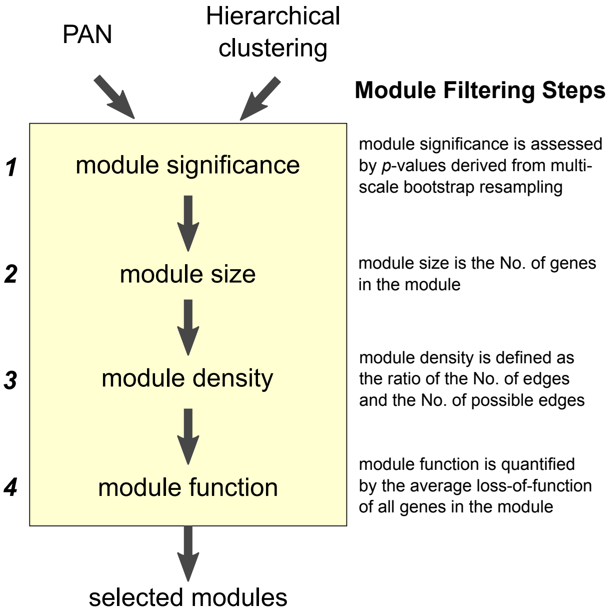
PAN
Hierarchical clustering
Module Filtering Steps
1
module significance
module significance is assessed by *p*-values derived from multi-scale bootstrap resampling
2
module size
module size is the No. of genes in the module
3
module density
module density is defined as the ratio of the No. of edges and the No. of possible edges
4
module function
module function is quantified by the average loss-of-function of all genes in the module
selected modules
