# Supplementary figures and images for: Posterior Association Networks and Functional Modules Inferred from Rich Phenotypes of Gene Perturbations
Source: PLoS Comput Biol. 2012 Jun 28;8(6):e1002566. doi: 10.1371/journal.pcbi.1002566 (PMC3386165; doi:10.1371/journal.pcbi.1002566)

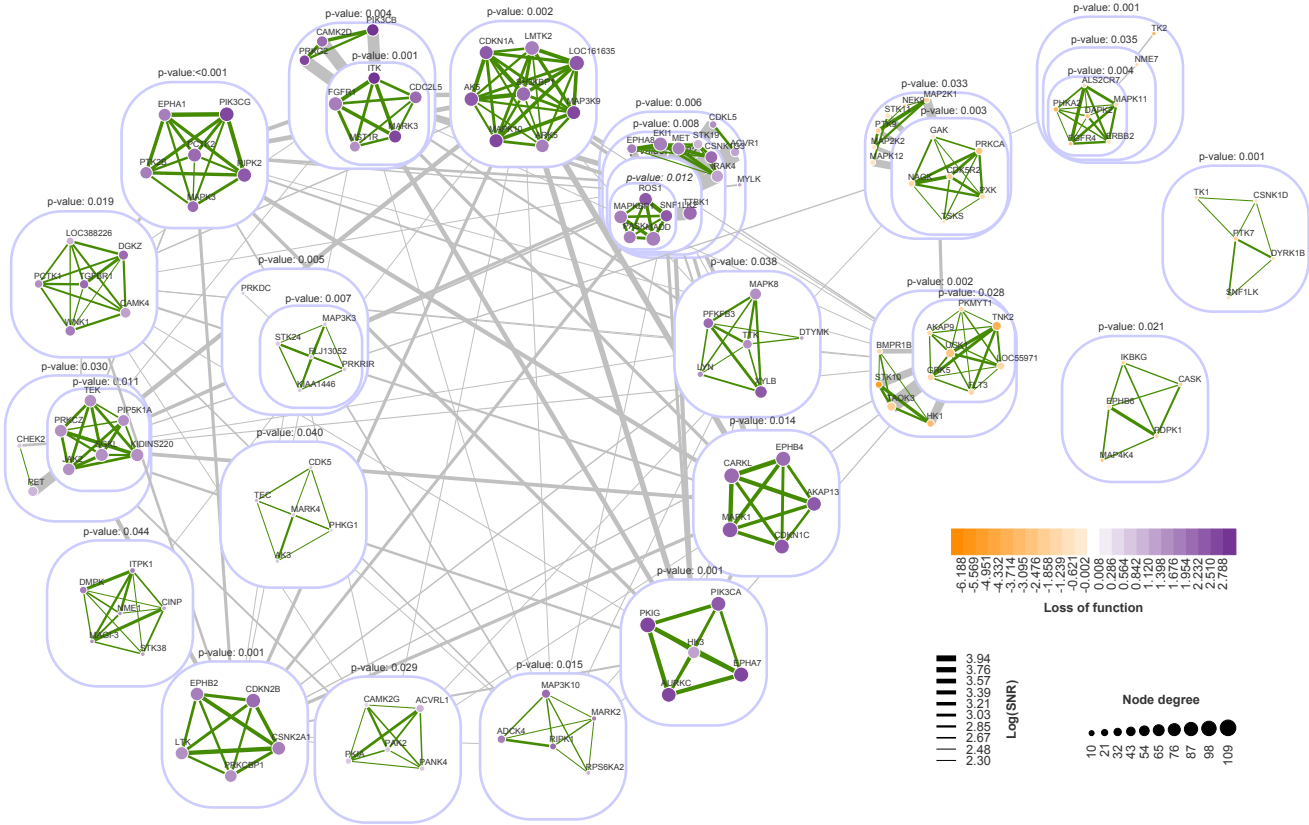

Supplement: Figure S2 — Predicted significant modules for Ewing's sarcoma. This figure is a more complete version for Figure 3(C). It includes also modules that are associated with positive loss of function (increased cancer cell viability) upon perturbation. The legends are the same as Figure 3(C) except that genes with positive perturbation phenotypes are colored in purple. (PDF) [file pcbi.1002566.s002.pdf]

**A**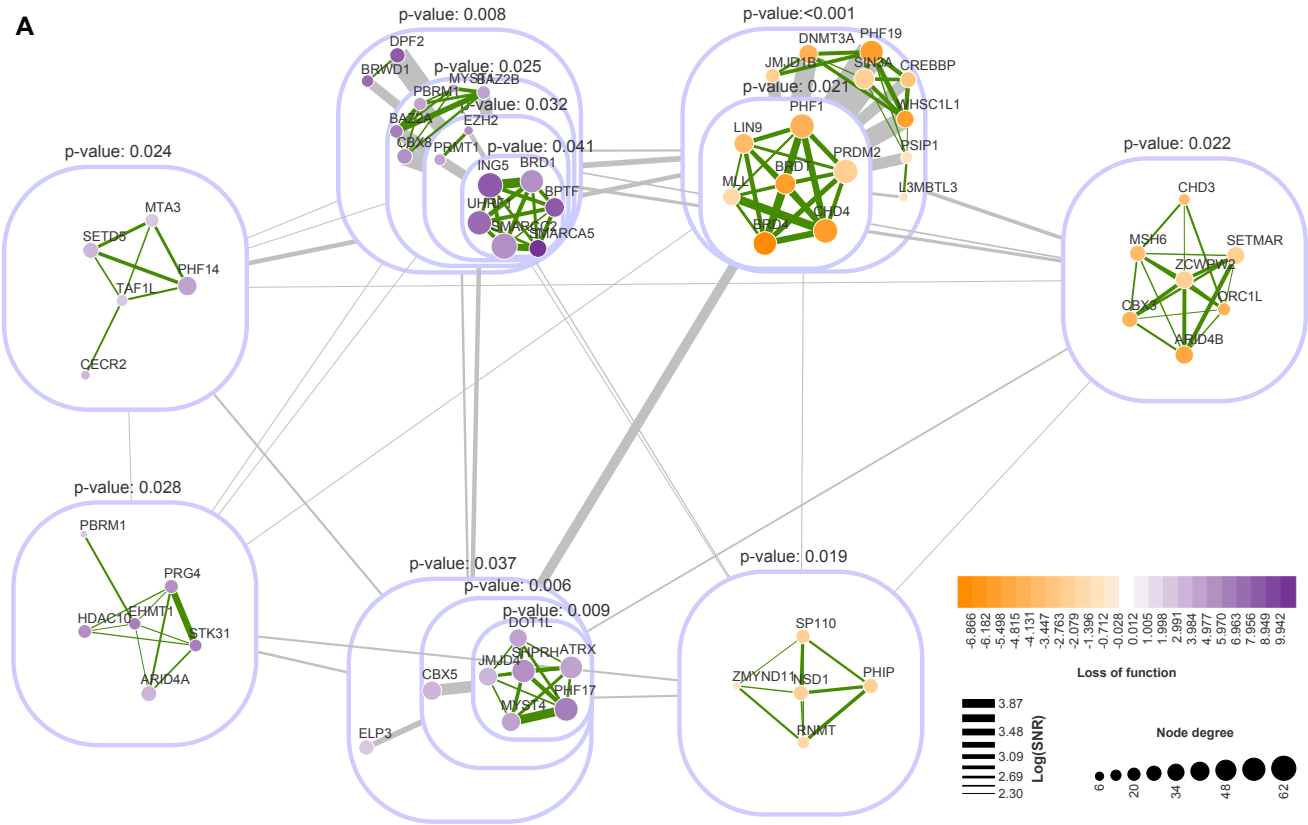

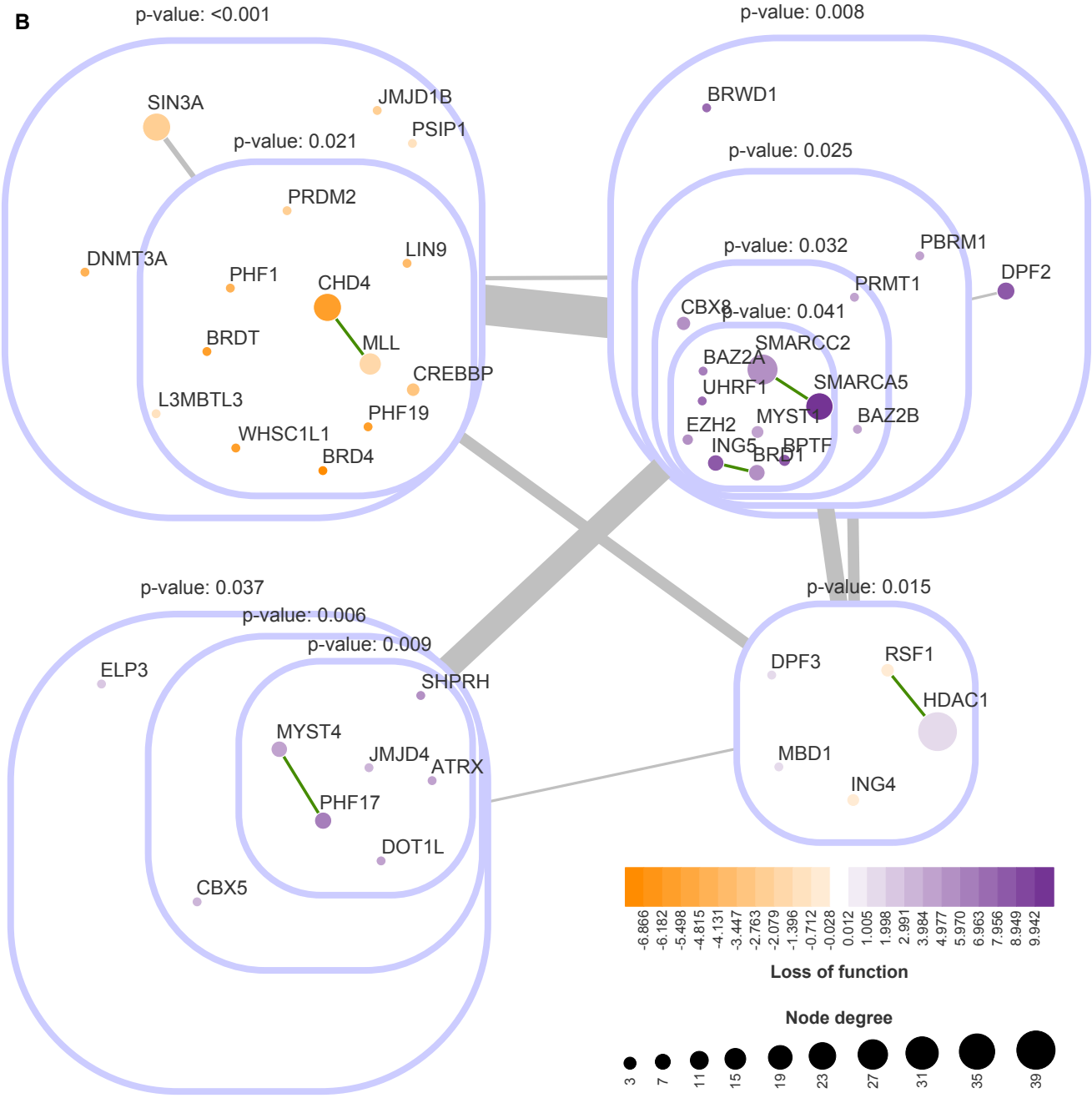

Supplement: Figure S3 — Predicted significant modules for epidermal stem cells. (A) The figure is a more complete version for Figure 7. It includes also modules that are associated with negative loss of function (decreased differentiation) upon perturbation. The legends are the same as Figure 7 except that genes with negative perturbation phenotypes are colored in orange. (B) The figure represent modules filtered using only the prior protein-protein interaction network. Ten modules in four root modules are obtained when filtering by a very baseline cutoff (). Using the same module density cutoff () as PAN, however, no significant module is obtained. The two figures suggest that the prior PPI network alone is not as informative as PAN in identifying functional modules. (PDF) [file pcbi.1002566.s003.pdf]
