# Supplementary material for: Posterior Association Networks and Functional Modules Inferred from Rich Phenotypes of Gene Perturbations
Source: PLoS Comput Biol. 2012 Jun 28;8(6):e1002566. doi: 10.1371/journal.pcbi.1002566 (PMC3386165; doi:10.1371/journal.pcbi.1002566)
Supplement: Table S1 — NO. of modules obtained at each filtering step using PAN or PPI for epidermal stem cells. (DOC) [file pcbi.1002566.s004.doc]

**Table S1. NO. of modules obtained at each filtering step using PAN or PPI for epidermal stem cells.**

|  | **No. of modules** | |
| --- | --- | --- |
| **filtering step** | **PAN** | **PPI** |
| All clusters | 331 | 331 |
| significant clusters (*p*-value < 0.05) | 90 | 90 |
| module size  (>= 5 and < half of the total No. of genes) | 39 | 39 |
| module density  ( > 0.5) | 13 | 0 |
| loss-of-function (average z-score < 0) | 9 | 0 |
| root modules | 4 | 0 |
