# Supplementary material for: Posterior Association Networks and Functional Modules Inferred from Rich Phenotypes of Gene Perturbations
Source: PLoS Comput Biol. 2012 Jun 28;8(6):e1002566. doi: 10.1371/journal.pcbi.1002566 (PMC3386165; doi:10.1371/journal.pcbi.1002566)
Supplement: Table S2 — Mapping between p -values and signal-to-noise ratios. (A) Mapping p-values to SNRs in the application to epidermal stem cells; (B) Mapping SNRs to p-values in the application to epidermal stem cells; (C) Mapping p-values to SNRs in the application to Ewing's sarcoma; (D) Mapping SNRs to p-values in the application to Ewing's sarcoma; (DOC) [file pcbi.1002566.s005.doc]

**Table S2. Translation between *p*-values and signal-to-noise ratios**

1. Translating *p*-values to SNRs in the application to epidermal stem cells

| ***p*-value** | **group** | **cosine similarity** | | **SNR** | |
| --- | --- | --- | --- | --- | --- |
|  |  | **lower tail** | **upper tail** | **lower tail** | **upper tail** |
| 0.05 | non-PPI | -0.63 | 0.63 | 1.50 | 1.51 |
|  | PPI | -0.63 | 0.63 | 4.27 | 4.16 |
| 0.01 | non-PPI | -0.80 | 0.80 | 4.97 | 4.82 |
|  | PPI | -0.80 | 0.80 | 14.17 | 13.29 |
| 0.001 | non-PPI | -0.91 | 0.91 | 11.49 | 11.47 |
|  | PPI | -0.91 | 0.91 | 32.79 | 31.63 |
| 1E-04 | non-PPI | -0.96 | 0.96 | 17.76 | 18.92 |
|  | PPI | -0.96 | 0.96 | 50.67 | 52.19 |

**(B) Translating SNRs to *p*-values in the application to epidermal stem cells**

| **SNR** | **group** | **cosine similarity** | | ***p*-value** | |
| --- | --- | --- | --- | --- | --- |
|  |  | **lower tail** | **upper tail** | **lower tail** | **upper tail** |
| 1 | non-PPI | -0.58 | 0.57 | 0.071 | 0.073 |
|  | PPI | -0.44 | 0.43 | 0.14 | 0.15 |
| 3 | non-PPI | -0.73 | 0.73 | 0.023 | 0.022 |
|  | PPI | -0.58 | 0.58 | 0.068 | 0.068 |
| 5 | non-PPI | -0.80 | 0.80 | 0.010 | 0.0093 |
|  | PPI | -0.65 | 0.66 | 0.043 | 0.041 |
| 10 | non-PPI | -0.89 | 0.89 | 1.68E-03 | 1.61E-03 |
|  | PPI | -0.75 | 0.76 | 1.803E-02 | 1.642E-02 |
| 30 | non-PPI | -0.99 | 0.99 | 5.58E-07 | 4.47E-06 |
|  | PPI | -0.90 | 0.90 | 1.40E-03 | 1.21E-03 |
| 100 | non-PPI | -1.00 | 1.00 | 7.43E-15 | 8.59E-11 |
|  | PPI | -1.00 | 0.99 | 6.78E-08 | 9.59E-07 |

**(C) Translating *p*-values to SNRs in the application to Ewing's sarcoma**

| ***p*-value** | **cosine similarity** | | **SNR** | |
| --- | --- | --- | --- | --- |
|  | **lower tail** | **upper tail** | **lower tail** | **upper tail** |
| 0.05 | -0.78 | 0.79 | 2.46 | 2.16 |
| 0.01 | -0.92 | 0.92 | 5.66 | 6.64 |
| 0.001 | -0.98 | 0.98 | 9.04 | 14.60 |
| 1E-04 | -1.00 | 1.00 | 11.48 | 24.95 |

(D) Translating SNRs to *p*-values in the application to Ewing's sarcoma

| **SNR** | **cosine similarity** | | ***p*-value** | |
| --- | --- | --- | --- | --- |
|  | **lower tail** | **upper tail** | **lower tail** | **upper tail** |
| 1 | -0.63 | 0.69 | 0.11 | 0.092 |
| 3 | -0.81 | 0.83 | 0.038 | 0.035 |
| 5 | -0.90 | 0.89 | 0.014 | 0.017 |
| 10 | -0.99 | 0.96 | 4.24E-04 | 3.59E-03 |
| 30 | -1.00 | 1.00 | 2.09E-10 | 4.17E-05 |
| 100 | -1.00 | 1.00 | 2.06E-11 | 1.08E-07 |
