# Supplementary material for: Posterior Association Networks and Functional Modules Inferred from Rich Phenotypes of Gene Perturbations
Source: PLoS Comput Biol. 2012 Jun 28;8(6):e1002566. doi: 10.1371/journal.pcbi.1002566 (PMC3386165; doi:10.1371/journal.pcbi.1002566)
Supplement: Table S3 — No. of modules obtained at each filtering step varying the SNR cutoff. (A) and (B) are for the application to epidermal stem cells and Ewing's sarcoma, respectively. (DOC) [file pcbi.1002566.s006.doc]

**Table S3. No. of modules obtained at each filtering step varying the SNR cutoff**

(A) Application to epidermal stem cells

|  | **No. of modules** | | | |
| --- | --- | --- | --- | --- |
| **filtering step** | **SNR=1** | **SNR=3** | **SNR=5** | **SNR=10** |
| All clusters | 331 | 331 | 331 | 331 |
| significant clusters (p-value < 0.05) | 90 | 90 | 90 | 90 |
| module size  (>= 5 and < half of the total No. of genes) | 39 | 39 | 39 | 39 |
| module density  ( > 0.5) | 28 | 22 | 20 | 13 |
| loss-of-function (average z-score > 0) | 21 | 16 | 15 | 9 |
| root modules | 12 | 9 | 8 | 4 |

(B) Application to Ewing’s sarcoma

|  | **No. of modules** | | | |
| --- | --- | --- | --- | --- |
| **filtering step** | **SNR=1** | **SNR=3** | **SNR=5** | **SNR=10** |
| All clusters | 571 | 571 | 571 | 571 |
| significant clusters (p-value < 0.05) | 304 | 304 | 304 | 304 |
| module size  (>= 5 and < half of the total No. of genes) | 65 | 65 | 65 | 65 |
| module density  ( > 0.5) | 65 | 57 | 51 | 30 |
| loss-of-function (average z-score < 0) | 26 | 25 | 23 | 9 |
| root modules | 18 | 17 | 15 | 5 |
